# The Associations between Pain Sensitivity and Knee Muscle Strength in Healthy Volunteers: A Cross-Sectional Study

**DOI:** 10.1155/2013/787054

**Published:** 2013-09-17

**Authors:** Marius Henriksen, Louise Klokker, Cecilie Bartholdy, Thomas Graven-Nielsen, Henning Bliddal

**Affiliations:** ^1^Clinical Motor Function Laboratory, The Parker Institute, Department of Rheumatology, Copenhagen University Hospital, Nordre Fasanvej 57, 2000 Frederiksberg, Denmark; ^2^Laboratory for Musculoskeletal Pain and Motor Control, Center for Sensory-Motor Interaction (SMI), Department of Health Science and Technology, Faculty of Medicine, Aalborg University, Fredrik Bajers Vej 7-D3, 9220 Aalborg, Denmark

## Abstract

*Objectives*. To investigate associations between muscle strength and pain sensitivity among healthy volunteers and associations between different pain sensitivity measures. *Methods*. Twenty-eight healthy volunteers (21 females) participated. Pressure pain thresholds (PPTs) were obtained from 1) computer-controlled pressure algometry on the vastus lateralis and deltoid muscles and on the infrapatellar fat pad and 2) computerized cuff pressure algometry applied on the lower leg. Deep-tissue pain sensitivity (intensity and duration) was assessed by hypertonic saline injections into the vastus lateralis, deltoid, and infrapatellar fat pad. Quadriceps and hamstring muscle strength was assessed isometrically at 60-degree knee flexion using a dynamometer. Associations between pain sensitivity and muscle strength were investigated using multiple regressions including age, gender, and body mass index as covariates. *Results*. Knee extension strength was associated with computer-controlled PPT on the vastus lateralis muscle. Computer-controlled PPTs were significantly correlated between sites (*r* > 0.72) and with cuff PPT (*r* > 0.4). Saline induced pain intensity and duration were correlated between sites (*r* > 0.39) and with all PPTs (*r* < −0.41). *Conclusions*. Pressure pain thresholds at the vastus lateralis are positively associated with knee extensor muscle strength. Different pain sensitivity assessment methods are generally correlated. The cuff PPT and evoked infrapatellar pain seem to reflect the general pain sensitivity. This trial is registered with ClinicalTrials.gov: NCT01351558.

## 1. Introduction

Musculoskeletal pain is very common. In Europe, as many as 1 out of 5 reports frequent and persistent musculoskeletal pain [1], and among those with musculoskeletal pain 50% have constant pain [2]. Thus, musculoskeletal pain is a significant challenge to society. While pain is the cardinal symptom in many musculoskeletal diseases, impaired muscle function is also a typical sign of musculoskeletal diseases, such as fibromyalgia and osteoarthritis [3, 4]. Adequate muscle function is crucial to maintain an independent life style, and reduced knee muscle strength is further associated with elevated risks of disability and mortality [5].

In recent years, many musculoskeletal diseases have been associated with increased pain sensitivity. Among patients with fibromyalgia, the increased pain sensitivity has been reported [6], and widespread increased pain sensitivity have also been demonstrated in subgroups of patients with osteoarthritis, lateral epicondylitis, and low back pain [7–9]. Jespersen et al. showed recently that the pain sensitivity on the lower leg assessed using cuff pressure algometry was associated with quadriceps muscle strength in fibromyalgia patients; patients with lower thigh muscle strength had higher pain sensitivity at the lower leg [10]. As muscle strength is a general marker of physical function, this finding suggests an association between physical function and generalized hyperalgesia in fibromyalgia patients. Whether that finding is specific for patients with fibromyalgia or whether it is a general physiological phenomenon is unknown. Also, it is unknown if pain sensitivity in other parts of the body, for example, upper extremity, is also associated with lower extremity muscle strength, or if such association is a local or regional phenomenon.

Computer-controlled pressure algometry and computerized cuff algometry are available and have been used to assess pain sensitivity in the past [11]. Pain sensitivity can also be assessed as the pain response to endogenous pain stimuli such as injections of algesic substances [12]. The model based on injection of hypertonic saline has been frequently used in experimental pain research and produced short lasting yet intense pain, referred pain sensations, and impaired sensorymotor functions [13, 14]. This type of pain may reflect the tonic pain sensitivity in contrast to the more phasic pain sensitivity assessed by estimating the pain thresholds. Mechanical and chemical modalities may excite different deep-tissue nociceptors and as such reflect different mechanisms [12], although this has not been investigated. Knowledge about the relationships between different pain sensitivity measures may aid interpretation and comparison between studies and methods.

The purpose of this study was to investigate the association between muscle strength and pain sensitivity among healthy volunteers. It was hypothesized that individuals with lower muscle strength would exhibit higher pain sensitivity. A secondary aim was to assess the associations between different pain sensitivity assessment measures.

## 2. Methods

### 2.1. Subjects

This study reports cross sectional (baseline) data from the randomized controlled trial *Exercise and Pain Sensitivity* that was prematurely terminated due to unsatisfactory recruiting and high dropout rate (clinicaltrials.gov: NCT01351558). Healthy volunteers between 18 and 35 years of age were recruited. Eligibility for participation was being in general good health. Exclusion criteria included history of musculoskeletal injuries or diseases, neurological, cardiovascular, or psychiatric diagnoses. All subjects gave informed consent. The study was performed according to the Declaration of Helsinki and approved by The Local Research Ethics Committee (ref: H-2-2011-031).

### 2.2. Assessment Protocol

In one session, subjects had their muscle strength assessed during knee flexion and extension. Subsequently, the pain sensitivity was assessed by saline-induced deep-tissue pain in the deltoid muscle (shoulder), vastus lateralis muscle (thigh), and infrapatellar fat pad (knee) and by mechanical pressure pain thresholds (PPTs) assessed by computer-controlled pressure algometry [11] and computerized cuff algometry [10]. 

### 2.3. Muscle Strength Assessment

Muscle strength of the quadriceps and hamstring muscles were assessed isometrically at 60° knee flexion by dynamometry (Biodex System3, Biodex Medical System, NY, USA). The dynamometer recorded the torque produced by muscle contractions. The protocol was comprised of 6 successive maximal efforts alternating between knee extension and knee flexion (3 in each direction). Each contraction lasted 5 seconds with a 10-second pause between them. The maximum peak torque value in each direction defined the muscle strength and is reported normalized to body mass (Nm/kg).

### 2.4. Saline-Induced Pain Sensitivity

Bolus injections of 1 mL sterile hypertonic saline (5.8%) were used to elicit experimental deep tissue pain. Injections were given into 2 muscles (vastus lateralis and deltoid) and 1 nonmuscle tissue (the infrapatellar fat pad). Injections were performed using a 2 mL plastic syringe with a disposable needle (27G). The experimental pain intensity was assessed on a 100 mm electronic visual analogue scale (VAS) with an external handheld slider to adjust the scale. The VAS was anchored with “no pain” and “maximum pain,” 0 mm and 100 mm, respectively. The VAS was collected for maximum 20 min. The maximal VAS score and the duration of the experimental pain were extracted from each injection. The pain duration was estimated as the difference between the first and the last time the VAS exceeded 0. If no pain was elicited (i.e., VAS = 0), the pain duration was set to 0.

### 2.5. Pressure Algometry

Computer-controlled pressure algometry (CCPA) was performed using a custom built pressure algometer controlled by a computer with dedicated custom software (Aalborg University, Denmark). PPTs were assessed at three assessment sites: m. vastus lateralis, m. deltoideus, and the infrapatellar fat pad. The CCPA applied mechanical stimuli perpendicular to the skin surface [11] using a round aluminum probe with a padded contact surface of 1 cm^2^ fixed to the tip of the piston. The pressure stimulation was feedback controlled via recordings of the actual force. The computer-controlled pressure stimulus was applied with an ascending pressure gradient of 59 kPa/s until the subject reported the first sensation of pain by pressing a push button. This recorded pressure defined the PPT_CCPA_. The PPT_CCPA_ of each assessment site was recorded three times. The time intervals between stimulations were individualized instructing the subjects to report when all sensation of the previous stimulation had vanished. The first PPT_CCPA_ was discarded to account for learning, and the two last PPT_CCPA_ values were averaged for further analysis.

### 2.6. Cuff Algometry

Computerized cuff algometry [15] consists of a tourniquet cuff (VBM Medizintechnik GmbH, Sulz, Germany) and a custom built computer-controlled air compressor (DoloCuff, Unique Electronic Aps, Denmark). The double chamber tourniquet cuff was wrapped around the lower leg at the bulky part of the gastrocnemius muscle. Both chambers of the cuff were automatically inflated simultaneously (compression rate: 1 kPa/s) until the subject reported the first sensation of pain by pressing a push-button. This recorded pressure defined the PPT_CUFF_. The PPT_CUFF_ was recorded three times separated by at least 60 seconds. The first PPT_CUFF_ was discarded to account for learning effects, and the two last PPT_CUFF_ values were averaged for further analysis.

### 2.7. Statistics

The associations between measures of pain sensitivity and muscle strength were assessed using univariate linear regressions with pain sensitivity measures as dependent variables and isometric knee muscle strength measures (extension and flexion) as independents variables. The regression analyses were repeated including age and gender as covariates. The associations between the different pain sensitivity measures were assessed by Pearson's product moment correlation coefficient. All analyses were done using SAS (9.2, Cary, NC, USA), and statistical significance was accepted at *P* < 0.05.

## 3. Results

Twenty-eight healthy subjects volunteered to participate in the study and their demographics are outlined in Table 1 together with descriptive summaries of the muscle strength and pain sensitivity tests. One subject did not experience pain during the experimental saline-induced pain sensitivity tests.

### 3.1. Muscle Strength and Pain Sensitivity

**Figure 1:**
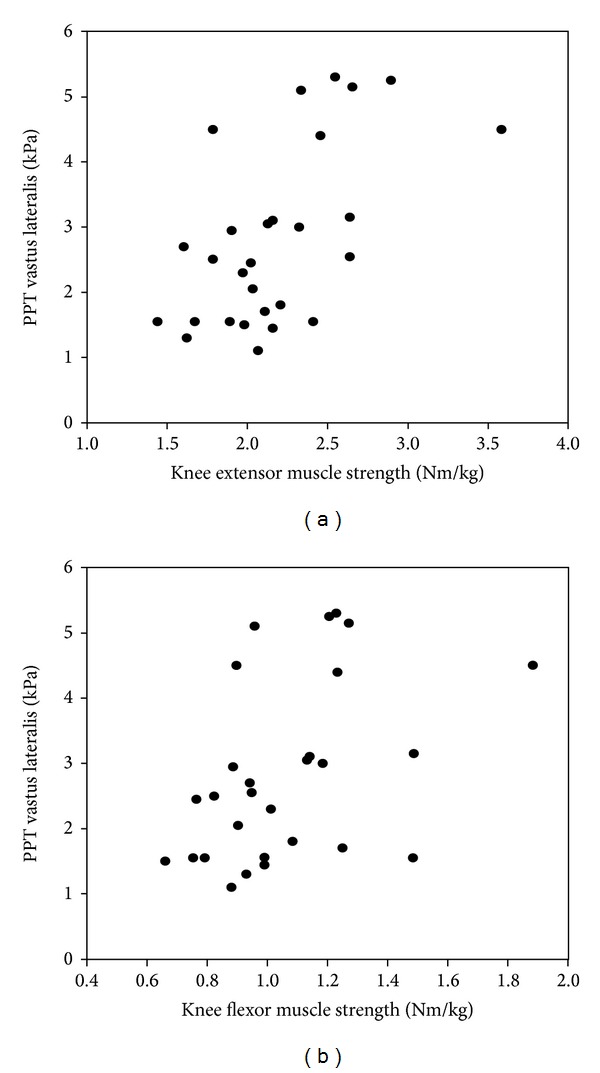
Scatter plots illustrating the association between isometric muscle strength in knee extension (a) and flexion (b) and pressure pain thresholds (PPT) in the vastus lateralis in healthy volunteers (*n* = 28).

Most pain sensitivity measures were not associated with muscle strength (Table 2), except for the PPT_CCPA_ at the infrapatellar fat pad and vastus lateralis muscle that were significantly positively associated with isometric knee extension muscle strength (univariate regression, Table 2). When adjusting for age, gender, and BMI, only the PPT_CCPA_ at vastus lateralis remained positively associated with isometric knee extension muscle strength (multivariate regression, Table 2). The PPT_CCPA_ at the vastus lateralis muscle was also significantly associated with isometric knee flexion muscle strength in the univariate regression, but this association was not significant when adjusting for covariates (Table 2).  Figure 1 illustrates the associations between muscle strength measures and PPT_CCPA_ at the vastus lateralis.

### 3.2. Correlation between Pain Sensitivity Assessment Methods

The PPT_CCPA_ at the three anatomical sites was mutually correlated and also correlated with the PPT_CUFF_ and the saline-induced pain intensity and duration in the infrapatellar fat pad, but not with saline-induced deltoid pain and duration (Table 3). The PPT_CUFF_ was significantly associated with intensity of the saline-induced pain at all three injection sites, but only with duration at the vastus lateralis (Table 3).

The saline-induced pain intensities were correlated across all three injection sites. The durations of saline-induced pain also correlated across sites. Moreover, the saline-induced pain intensities and durations correlated for injections into the vastus lateralis and the infrapatellar fat pad. The pain intensity after injections into the infrapatellar fat pad correlated also with the duration of pain after injections into the vastus lateralis muscle and the infrapatellar fat pad. 

## 4. Discussion

This study shows for the first time a significant association between pressure pain sensitivity in the vastus lateralis muscle and isometric knee extensor muscle strength among healthy volunteers when adjusting for age, sex, and body mass index. Further, this study demonstrates that different pain sensitivity assessment parameters are correlated especially within the same modality.

### 4.1. Muscle Strength and Pain Sensitivity

Previously, Jespersen et al. demonstrated a correlation between cuff pressure pain sensitivity at the lower leg and knee extensor and flexor muscle strength among patients with fibromyalgia [10]. In the present study, the same correlation was however not demonstrated among healthy subjects, which indicate that the findings in Jespersen et al. are specific for patients with chronic pain. This may be explained by the prevailing theory that chronic pain conditions are associated with sensitization of the pain system in parallel with reduced physical function. In the present study, only healthy volunteers were included and since these are not sensitized, this may explain the lack of correlation. 

An association between the vastus lateralis pressure pain sensitivity and knee extensor muscle strength was demonstrated. This indicates that persons with higher muscle strength exhibit less sensitivity to pressure pain in the same muscle. No associations between the other pain sensitivity measures and knee muscle strength were found which may suggest that in healthy subjects, muscle function is only associated with the pressure pain sensitivity in the local muscle tissue. It could be speculated that this tight coupling may be disturbed in painful conditions, and the association between muscle function and pain sensitivity becomes marked. This would be supported by the findings in Jespersen et al. [10]. In that study, knee muscle strength was associated with pain sensitivity in a neighboring area (the lower leg), indicating that the association between pain sensitivity and muscle function has developed from a localized phenomenon in the healthy subjects (as in the present study) to a more generalized phenomenon in patients with fibromyalgia (as in Jespersen et al. [10]). This would be in line with the suggested gradual spatial spreading of sensitization and hyperalgesia in chronic musculoskeletal pain [16] and its association with a concomitant gradual spreading of reduced physical function, from localized single muscle impairment to generalized decreased motor function and physical disablement. However, the chronology and causal relationships remain unsolved in the parallel development of these phenomena and need to be a focus in future research. While saline-induced pain sensitivity was not associated with muscle strength in the present study, the local pressure pain sensitivity was which could inform a hypothesis that pressure pain sensitivity can be affected by interventions targeting muscle strength, potentially explaining parts of the analgesic effects of exercise in chronic musculoskeletal pain conditions. 

An alternative explanation of the relation between strength and pressure pain sensitivity is related to the accommodation of nociceptors to be excited by the pressure stimulation. Recently, it was demonstrated that a stronger pressure was needed to evoke the same intramuscular biomechanical strain in muscles producing higher contraction levels compared with lower contraction levels resulting in higher pain thresholds for muscles that are contracted harder [17]. Thus, the higher pressure pain thresholds may be related with the stronger muscle as demonstrated in this present study.

Implications of the present findings are difficult to infer due to the cross-sectional study design. However, the results may suggest that exercise improving strength have beneficial effects on the pain sensitivity, which could be of relevance to patients with chronic musculoskeletal diseases associated with hyperalgesia. Among healthy subjects, the results may suggest that reduced pain sensitivity in stronger muscles impact the sensation of pain and discomfort during contact sports and perceived pain severity upon sustained muscle injuries. However, this remains speculative and needs further studies.

### 4.2. Correlation between Pain Sensitivity Assessment Methods

The present study demonstrated the concurrence between different pain sensitivity assessment methods. The results shows that pain sensitivity measurements related to the lower extremities were generally correlated, indicating that these assessment methods are comparable. The results also show that there is a variability in the PPT_CCPA_ between the different sites (Table 1). This may be explained by differences in the tissue hardness between the sites because higher pain thresholds have been found for harder muscles compared with soft muscle structures [17]. The computerized cuff pressure algometry and the saline-induced pain in the infrapatellar fat pad showed statistically significant association between pain sensitivity assessment at the lower leg and PPTs and experimental pain intensities in the deltoid muscle. This indicates that both methods applied to the lower extremity may be useful as an assessment of the overall pain sensitivity. 

## 5. Conclusion

Localized pressure pain thresholds at the vastus lateralis are positively associated with knee extensor muscle strength. Different means of assessing pain sensitivity are generally correlated, especially within a modality. Interestingly, the cuff pain thresholds and saline-induced infrapatellar fat pad pain seems to reflect the general pain sensitivity.

## Figures and Tables

**Table 1:** Demographics of subjects and summaries of pain sensitivity assessments. Data are given as mean, standard deviation (SD), minimum, and maximum unless otherwise indicated.

	Mean	(SD)	Min.	Max.
Age, years	26.8	(4.8)	18.8	35.6
Height, cm	170.8	(7.0)	156.0	184.0
Weight, kg	72.9	(11.2)	53.0	98.8
Body mass index, kg/m^2^	24.9	(3.3)	18.3	32.6
Female/male sex, *n* (%)	21/7	(75/25%)	—	—
Isometric muscle strength				
Knee Extension, Nm/kg	2.2	(0.4)	1.4	3.6
Knee Flexion, Nm/kg	1.1	(0.3)	0.7	1.9
PPT_CCPA_				
Vastus Lateralis, kPa	275	(128)	137	804
Deltoideus, kPa	294	(196)	29	765
Infrapatellar fat pad, kPa	402	(186)	137	801
PPT_CUFF_ lower leg, kPa	20.9	(6.6)	10.3	36.7
Experimental pain intensity				
Vastus Lateralis, mm	39.6	(24.2)	0.0	100.0
Deltoideus, mm	33.6	(19.5)	0.0	77.0
Infrapatellar fat pad, mm	50.3	(27.3)	0.0	100.0
Experimental pain duration				
Vastus Lateralis, s	553.1	(195.9)	0.0	1099.0
Deltoideus, s	462.8	(239.9)	0.0	1100.0
Infrapatellar fat pad, s	727.7	(278.5)	0.0	1200.0

PPT_CCPA_: pressure pain threshold assessed by computer-controlled pressure algometry; PPT_CUFF_: pressure pain threshold assessed by computerized cuff algometry.

**Table 2:** Relationships between isometric knee muscle strength in extension and flexion and different measures of pain sensitivity at different assessment sites.

	Univariate analyses		Multivariate analyses^†^	
	Coefficient (95% CI)	*P*	Coefficient (95% CI)	*P*
PPT_CCPA _(kg)				
Vastus Lateralis				
Knee extension (Nm/kg)	1.77 (0.79; 2.75)	0.001	1.20 (0.21; 2.19)	0.02
Knee flexion (Nm/kg)	2.21 (0.33; 4.10)	0.02	1.50 (−0.31; 3.32)	0.10
Deltoideus				
Knee extension (Nm/kg)	1.40 (−0.32; 3.12)	0.10	0.48 (−1.29; 2.25)	0.58
Knee flexion (Nm/kg)	1.30 (−1.76; 4.36)	0.39	0.21 (2.87; 3.28)	0.89
Infrapatellar fat pad				
Knee extension (Nm/kg)	1.73 (0.12; 3.33)	0.04	1.61 (−0.35; 3.47)	0.09
Knee flexion (Nm/kg)	0.91 (−2.07; 3.89)	0.53	0.59 (−2.82; 4.00)	0.72
PPT_CUFF _lower leg (kPa)				
Knee extension (Nm/kg)	0.70 (−5.49; 6.88)	0.82	−0.23 (−7.56; 7.09)	0.95
Knee flexion (Nm/kg)	−0.26 (−10.89; 10.36)	0.96	−0.66 (−13.3; 12.0)	0.92
Saline-induced pain intensity (mm)				
Vastus Lateralis				
Knee extension (Nm/kg)	−6.59 (−26.55; 13.38)	0.50	−4.29 (−27.30; 18.71)	0.70
Knee flexion (Nm/kg)	−3.28 (−37.81; 31.25)	0.85	−4.23 (−44.08; 35.63)	0.83
Deltoideus				
Knee extension (Nm/kg)	5.17 (−12.76; 23.11)	0.56	−0.60 (−21.85; 20.64)	0.95
Knee flexion (Nm/kg)	6.19 (−24.68; 37.06)	0.68	−7.12 (−43.73; 29.50)	0.69
Infrapatellar fat pad				
Knee extension (Nm/kg)	−13.00 (−36.81; 10.81)	0.27	−6.13 (−32.51; 20.24)	0.64
Knee flexion (Nm/kg)	−11.94 (−53.48; 29.61)	0.56	−7.56 (−53.26; 38.15)	0.74
Experimental pain duration (s)				
Vastus Lateralis				
Knee extension (Nm/kg)	−151.3 (−322.7; 20.16)	0.08	−89.5 (−285.2; 106.3)	0.35
Knee flexion (Nm/kg)	−197.3 (−499.1; 104.5)	0.19	−132.6 (−472.7; 207.5)	0.43
Deltoideus				
Knee extension (Nm/kg)	−92.4 (−312.9; 128.2)	0.40	−112.2 (−381.4; 155.9)	0.40
Knee flexion (Nm/kg)	−265.4 (−634.0; 103.1)	0.15	−323.4 (−775.0; 128.2)	0.15
Infrapatellar fat pad				
Knee extension (Nm/kg)	−221.5 (−453.7; 10.7)	0.06	−105.8 (361.4; 149.7)	0.40
Knee flexion (Nm/kg)	−211.2 (−629.4; 207.0)	0.31	−42.6 (−490.7; 405.6)	0.85

^†^Adjusting for age and sex.

PPT_CCPA_: pressure pain threshold assessed by computer-controlled pressure algometry; PPT_CUFF_: pressure pain threshold assessed by computerized cuff algometry.

**Table 3:** Correlation matrix between different measures of pain sensitivity at different assessment sites. Correlation coefficients (Pearson) at the top with the *P* value below. Statistically significant correlation coefficients are marked with bold.

			Pressure pain thresholds				Saline-induced pain					
			CCPA			CUFF	Max. intensity (mm)			Duration (s)		
			VL	DELT	IPFP	LL	VL	DELT	IPFP	VL	DELT	IPFP
Pressure pain thresholds	CCPA	VL	**1**									
			**—**									
		DELT	**0.88**	**1**								
			**<0.0001**	**—**								
		IPFP	**0.72**	**0.78**	**1**							
			**<0.0001**	**<0.0001**	**—**							
	CUFF	LL	**0.40**	**0.55**	**0.53**	**1**						
			**0.04**	**0.003**	**0.004**	**—**						

Saline-induced pain	Max. Intensity (mm)	VL	−0.37	−**0.37**	−**0.49**	−**0.49**	**1**					
			0.06	**0.05**	**0.008**	**0.009**	**—**					
		DELT	−0.19	−0.21	−0.26	−**0.55**	**0.76**	**1**				
			0.33	0.29	0.18	**0.02**	**<0.0001**	**—**				
		IPFP	−**0.54**	−**0.64**	−**0.61**	−**0.41**	**0.63**	**0.44**	**1**			
			**0.003**	**0.0002**	**0.0006**	**0.03**	**0.0003**	**0.02**	**—**			
	Duration (s)	VL	−**0.47**	−**0.53**	−**0.48**	−**0.40**	**0.52**	0.28	**0.41**	**1**		
			**0.01**	**0.003**	**0.01**	**0.03**	**0.005**	0.15	**0.03**	**—**		
		DELT	−0.25	−0.23	−0.15	−0.16	0.16	0.29	0.15	**0.39**	**1**	
			0.21	0.23	0.46	0.42	0.43	0.14	0.43	**0.04**	**—**	
		IPFP	−**0.60**	−**0.66**	−**0.56**	−0.37	0.30	0.22	**0.60**	**0.52**	**0.39**	**1**
			**0.0008**	**0.0001**	**0.002**	0.055	0.12	0.26	**0.0007**	**0.005**	**0.04**	**—**

CCPA: computer-controlled pressure algometry. CUFF: computerized cuff algometry. VL: m. vastus lateralis.; DELT: m. deltoideus; IPFP: infrapatellar fat pad; LL: lower leg.

## References

[1] Breivik H, Collett B, Ventafridda V, Cohen R, Gallacher D (2006). Survey of chronic pain in Europe: prevalence, impact on daily life, and treatment. *European Journal of Pain*.

[2] Woolf AD, Zeidler H, Haglund U (2004). Musculoskeletal pain in Europe: its impact and a comparison of population and medical perceptions of treatment in eight European countries. *Annals of the Rheumatic Diseases*.

[3] Henriksen M, Lund H, Christensen R (2009). Relationships between the fibromyalgia impact questionnaire, tender point count, and muscle strength in female patients with fibromyalgia: a cohort study. *Arthritis Care and Research*.

[4] Slemenda C, Heilman DK, Brandt KD, Katz BP, Mazzuca SA, Braunstein EM (1998). Reduced quadriceps strength relative to body weight: a risk factor for knee osteoarthritis in women?. *Arthritis & Rheumatism*.

[5] Rantanen T (2003). Muscle strength, disability and mortality. *Scandinavian Journal of Medicine and Science in Sports*.

[6] Kosek E, Ekholm J, Hansson P (1995). Increased pressure pain sensibility in fibromyalgia patients is located deep to the skin but not restricted to muscle tissue. *Pain*.

[7] Arendt-Nielsen L, Nie H, Laursen MB (2010). Sensitization in patients with painful knee osteoarthritis. *Pain*.

[8] Fernández-Carnero J, Fernández-De-Las-Peñas C, De La Llave-Rincón AI, Ge H-Y, Arendt-Nielsen L (2009). Widespread mechanical pain hypersensitivity as sign of central sensitization in unilateral epicondylalgia a blinded, controlled study. *Clinical Journal of Pain*.

[9] O’Neill S, Kjær P, Graven-Nielsen T, Manniche C, Arendt-Nielsen L (2011). Low pressure pain thresholds are associated with, but does not predispose for, low back pain. *European Spine Journal*.

[10] Jespersen A, Dreyer L, Kendall S (2007). Computerized cuff pressure algometry: a new method to assess deep-tissue hypersensitivity in fibromyalgia. *Pain*.

[11] Graven-Nielsen T, Mense S, Arendt-Nielsen L (2004). Painful and non-painful pressure sensations from human skeletal muscle. *Experimental Brain Research*.

[12] Graven-Nielsen T (2006). Fundamentals of muscle pain, referred pain, and deep tissue hyperalgesia. *Scandinavian Journal of Rheumatology*.

[13] Henriksen M, Alkjær T, Lund H (2007). Experimental quadriceps muscle pain impairs knee joint control during walking. *Journal of Applied Physiology*.

[14] Tucker KJ, Hodges PW (2009). Motoneurone recruitment is altered with pain induced in non-muscular tissue. *Pain*.

[15] Polianskis R, Graven-Nielsen T, Arendt-Nielsen L (2001). Computer-controlled pneumatic pressure algometry—a new technique for quantitative sensory testing. *European Journal of Pain*.

[16] Graven-Nielsen T, Arendt-Nielsen L (2010). Assessment of mechanisms in localized and widespread musculoskeletal pain. *Nature Reviews Rheumatology*.

[17] Finocchietti S, Mørch CD, Arendt-Nielsen L, Graven-Nielsen T (2011). Effects of adipose thickness and muscle hardness on pressure pain sensitivity. *Clinical Journal of Pain*.

